# Transesophageal echocardiographic evaluation of an intraoperative retrograde acute aortic dissection: case report

**DOI:** 10.1186/1476-7120-4-19

**Published:** 2006-04-03

**Authors:** William C Culp, Karen J Morgan-Vanderlick, Charles G Reiter

**Affiliations:** 1 Department of Anesthesiology, Scott & White Hospital, The Texas A&M University System Health Science Center College of Medicine, Temple, Texas, USA; 2 Division of Cardiothoracic Surgery, Scott & White Hospital, The Texas A&M University System Health Science Center College of Medicine, Temple, Texas, USA

## Abstract

**Background:**

We report an intraoperative retrograde dissection of the aorta and its subsequent evaluation by transesophageal echocardiography (TEE).

**Case presentation:**

A 78 year old woman with an ascending aortic aneurysm without dissection and coronary artery disease was brought to the operating room for aneurysm repair and coronary artery bypass grafting. After initiation of cardiopulmonary bypass through a femoral artery cannula, aortic dissection was noted and subsequently imaged by TEE.

**Conclusion:**

Retrograde aortic dissection through the femoral artery is life-threatening. Intraoperative TEE can be used to diagnose this uncommon event, and should be considered after initiation of bypass.

## Background

Aortic dissection is a life-threatening illness that requires early diagnosis to allow the greatest chance of survival [1], and is a known complication of aortic and femoral cannulation as well as cardiac and aortic surgeries. We report a case of acute intraoperative retrograde aortic dissection after femoral artery cannulation that occurred during replacement of an aneurysmal ascending aorta. This dissection was evaluated promptly by use of intraoperative transesophageal echocardiography (TEE). The role of intraoperative TEE can be expanded to include assessment for acute aortic dissection during procedures requiring femoral cannulation.

## Case presentation

A 78 year old woman weighing 47 kg and 160 cm tall was scheduled for ascending aortic aneurysm repair and coronary artery bypass grafting. Her past medical history was remarkable for smoking, hypertension, peripheral vascular disease, and prior myocardial infarction. Magnetic resonance imaging (MRI) revealed an ascending aortic aneurysm with a maximum diameter of 6.6 cm. The descending aorta was dilated to 3.9 cm, but returned to a more normal diameter of 2.4 cm at the level of the diaphragmatic hiatus. No intimal flap was present to suggest dissection. Cardiac catheterization was performed by accessing the right femoral artery with a 6 F introducer sheath using a small skin incision followed by blunt dissection. The right coronary artery was 70% stenosed, the left circumflex had a 90% stenosis, and the left anterior descending artery was 50% stenosed. Moderate aortic insufficiency and a large ascending aortic aneurysm measuring 6.8 cm were noted. No dissection was seen.

**Figure 1:**
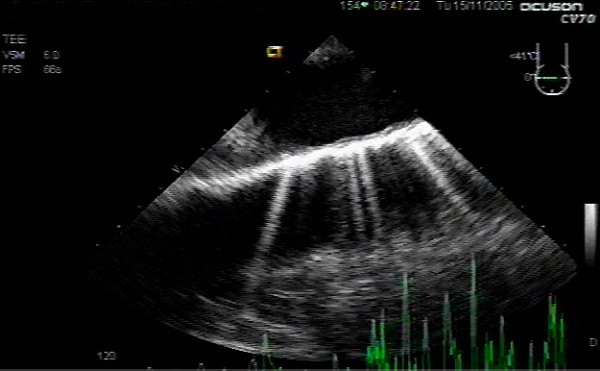
Upper esophageal aortic arch long axis view pre-cardiopulmonary bypass, demonstrating mild intimal thickening and irregularity and generalized large aortic diameter, but no dissection.

**Figure 2:**
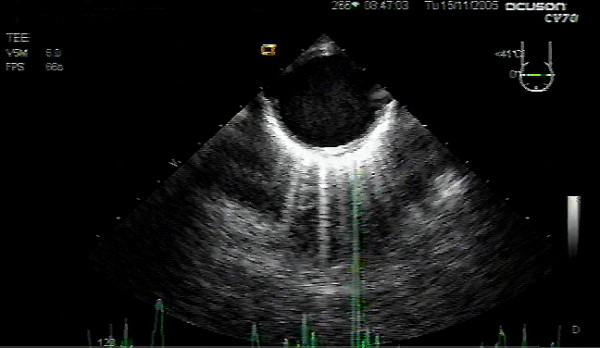
Descending aortic short axis view demonstrating mild aortic enlargement.

The patient was brought to the operating room where after placing a right brachial arterial catheter, general endotracheal anesthesia was induced with etomidate and succinylcholine and maintained with fentanyl, midazolam, isoflurane, and pancuronium. An 8.5 Fr percutaneous introducer sheath was placed in the left subclavian vein and a Swan-Ganz catheter advanced into the pulmonary artery. Multiplane TEE was performed (Acuson CV70 and V5M probe, Siemens Medical Solutions USA, Inc., Malvern, PA) which confirmed the cardiac catheterization and MRI findings of ascending aortic aneurysm. No dissection was seen (Figures 1 and 2). Moderate aortic insufficiency and mild systolic dysfunction were noted. The patient was heparinized. Using a #15 scalpel blade, the left common femoral artery was exposed, incised, and then cannulated under direct vision using a 20 Fr femoral arterial cannula 25 cm in length (Fem-Flex Arterial Cannula, Edwards Life Science, Irvine, CA). Good blood return was noted from the cannula immediately after placement. A volume of 100 mL of crystalloid was then administered by the perfusionist to assess the adequacy of this line. The femoral line pressure was noted to be approximately 200 mmHg, higher than typical line pressures, but attributed to the relatively small cannula size. Venous cannulae were then placed in the superior and inferior vena cava. Cardiopulmonary bypass was initiated with a flow of 2.2 cardiac index (CI) and systemic blood pressure of 70 mmHg, with femoral line pressures near 200 mmHg. The patient was not hypertensive at any point in the pre-bypass stage.

Deep hypothermic circulatory arrest was initiated and the aortic aneurysm incised and resected. A dissection flap within the aneurysm was noted, extending nearly to the level of the aortic annulus. A Dacron graft was then attached to the superior ascending aorta, and cardiopulmonary bypass re-initiated. Blood pressure was extremely low as measured by the brachial arterial catheter despite flow of 2.2 CI. The aorta was then fenestrated, restoring normal blood pressure. The inferior aspect of the graft was then anastamosed to the aortic annulus after buttressing the annulus with felt. Coronary artery bypass grafting was then performed. Cardiopulmonary bypass lasted 162 minutes, with a circulatory arrest time of 24 minutes.

**Figure 3:**
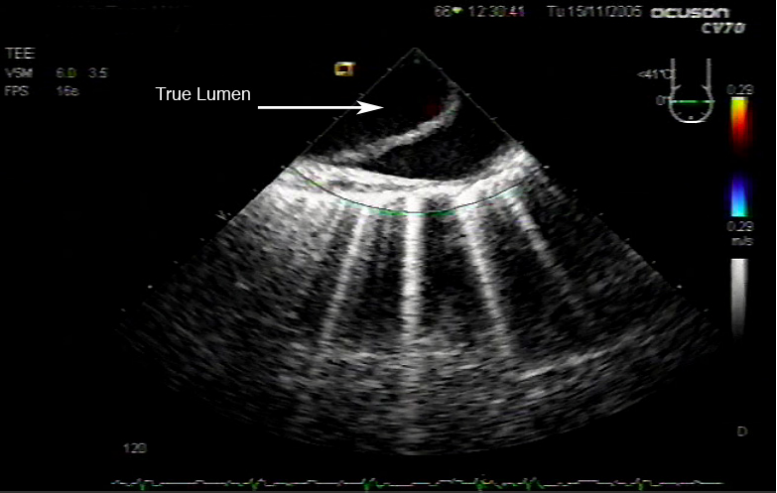
Upper esophageal aortic arch long axis view post-cardiopulmonary bypass, demonstrating the proximal portion of the aortic dissection.

**Figure 4:**
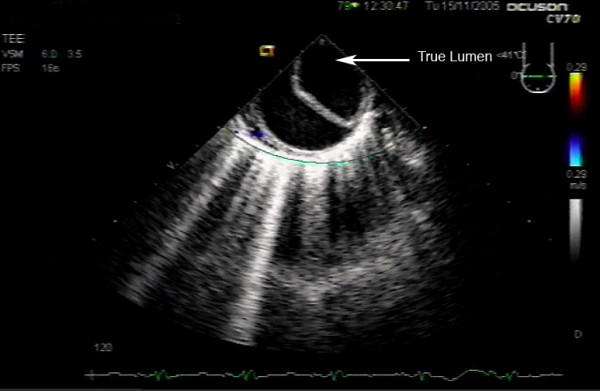
Descending aortic short axis view post-cardiopulmonary bypass demonstrating aortic dissection.

Separation from bypass was achieved using epinephrine and calcium. TEE examination revealed a now competent aortic valve, but clearly demonstrated a large aortic dissection from the aortic arch down more than 20 cm into the thoracoabdominal aorta, extending pass the imaging depth of TEE. No entry site was visualized (Figures 3 and 4). The patient was hemodynamically stable, and was subsequently closed. Approximately 20 minutes after closure, the patient became profoundly hypotensive and the ventricles fibrillated. Aggressive resuscitation was provided with amiodarone, epinephrine, and electrical cardioversion while the chest was opened and bypass reinstituted. The incision was extended to expose the abdominal viscera which appeared hypoperfused. There was no resumption of cardiac function despite good flow on bypass, the patient was anuric and profoundly acidemic. Bypass was discontinued and the patient declared dead. Autopsy was declined.

Aortic dissection is a relatively rare intraoperative event, occurring in 0.16% of operations involving aortic cannulation and cardiopulmonary bypass [2]. Acute aortic dissection may also occur in a retrograde fashion when other vessels, such as the femoral, axillary, or carotid arteries are cannulated [3-5]. The incidence of retrograde dissection as a result of femoral arterial cannulation has been reported as 0.2–1.3%, and is likely caused by either direct (cannula) or indirect (jet) trauma to the vessel or atherosclerotic plaque [6,7]. Risk factors for dissection include hypertension, smoking, atherosclerosis, connective tissue disorders, and high cannula jet velocities. Aortic dissection is often lethal, but prompt diagnosis and treatment can result in improved survival. Femoral arterial cannulation for cardiopulmonary bypass is standard practice for many aortic surgeries and may become even more common as minimally invasive cardiac surgeries proliferate. As a result, physicians should be aware of the risk of dissection in at-risk patient populations.

In our patient, we suspect that femoral cannulation led to an ilioaortic dissection which advanced retrograde up the length of the aorta as cardiopulmonary bypass was begun. Surgical exposure of the ascending aorta 30 minutes after initiation of bypass revealed the intimal flap, and then the extent of the dissection was evaluated by TEE. Immediate TEE examination of the descending aorta upon initiation of bypass would likely have shown the aortic dissection in evolution. This could have prompted cessation of flow into the femoral cannula and perhaps decreased the extent of the dissection. The management plan after chest closure was for either medical therapy or endovascular stent placement after the patient stabilized, however, an acute ischemic event likely was the cause of the patient's sudden cardiac death before definitive dissection therapy could be provided.

TEE is one of several highly sensitive and specific imaging modalities for assessment of aortic dissection. The sensitivity and specificity for TEE, computed axial tomography (CT), and MRI in one study was 100%/94%, 100%/100%, and 100%/94% respectively [8]. In contrast to CT, MRI, and aortography, TEE is mobile, rapid, and can be used intraoperatively. Color Doppler techniques also permit flow studies that can determine flow within true and false lumens and help delineate thrombosed lumens from those with active blood flow. Further, there is early evidence that TEE can be used to assess malperfusion or dissection of the arterial supply of the abdominal viscera [9]. This test is not perfect, however, as false-positive findings have been described with femoral arterial bypass cannulae in the setting of pseudodissection phenomenon. This is characterized by transient echogenic "smoke" and a pseudo false lumen in the descending aorta lasting about 30 seconds after initiation of bypass [10]. Additionally, TEE is unable to image the distal ascending aorta and proximal aortic arch well due to tracheal interposition. Overall accuracy for echocardiographic diagnosis of aortic dissection is very high and is comparable to other imaging modalities.

## Conclusion

We report a case of acute intraoperative retrograde aortic dissection after femoral arterial cannulation that was evaluated by intraoperative TEE. Physicians should be aware of this potentially life-threatening complication and echocardiographic evaluation of the aorta should be considered in patients after femoral cannulation as cardiopulmonary bypass is initiated.

## Competing interests

The author(s) declare that they have no competing interests.

## Authors' contributions

WCC supervised the TEE and wrote the paper.

KJM assisted with the TEE and draft revisions.

CGR assisted with the draft and performed the operation.
